# Supplementary material for: Plasmonic nanostar photocathodes for optically-controlled directional currents
Source: Nat Commun. 2020 Mar 13;11:1367. doi: 10.1038/s41467-020-15115-0 (PMC7069989; doi:10.1038/s41467-020-15115-0)
Supplement: Supplementary file 1 — Supplementary Information [file 41467_2020_15115_MOESM1_ESM.pdf]

Supplementary Information

Plasmonic Nanostar Photocathodes for Optically-Controlled Directional Currents

Pettine *et al.*

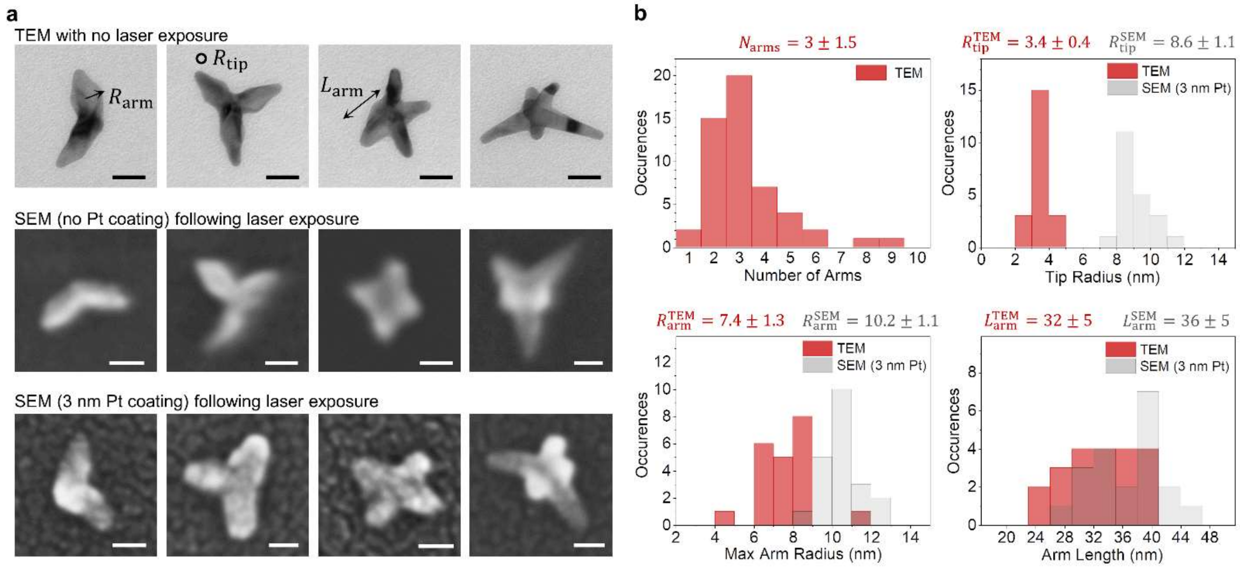

Supplementary Fig. 1 | Nanostar statistical characterization via electron microscopy. a Example transmission electron microscopy (TEM) and scanning electron microscopy (SEM) images of stars with different numbers of arms, N_{arms}. All SEM micrographs are collected following exposure to laser intensities of $\sim$0.1 GW cm^{-2} or greater in photoemission studies. The lack of observable morphological differences between the unexposed nanostars in TEM images and the laser-exposed nanostars in SEM images (those with no Pt coating, specifically) provides strong evidence that no appreciable particle melting occurs during photoemission studies. For most studies, a 3 nm Pt coating was applied to the nanostars to improve SEM contrast and thereby clarify the nanostar geometries. All scale bars are 25 nm. **b** Statistical characterization of nanostar dimensions in TEM and SEM (3 nm Pt coating) images. An approximate 3 nm broadening is evident in all features due to the Pt coating.

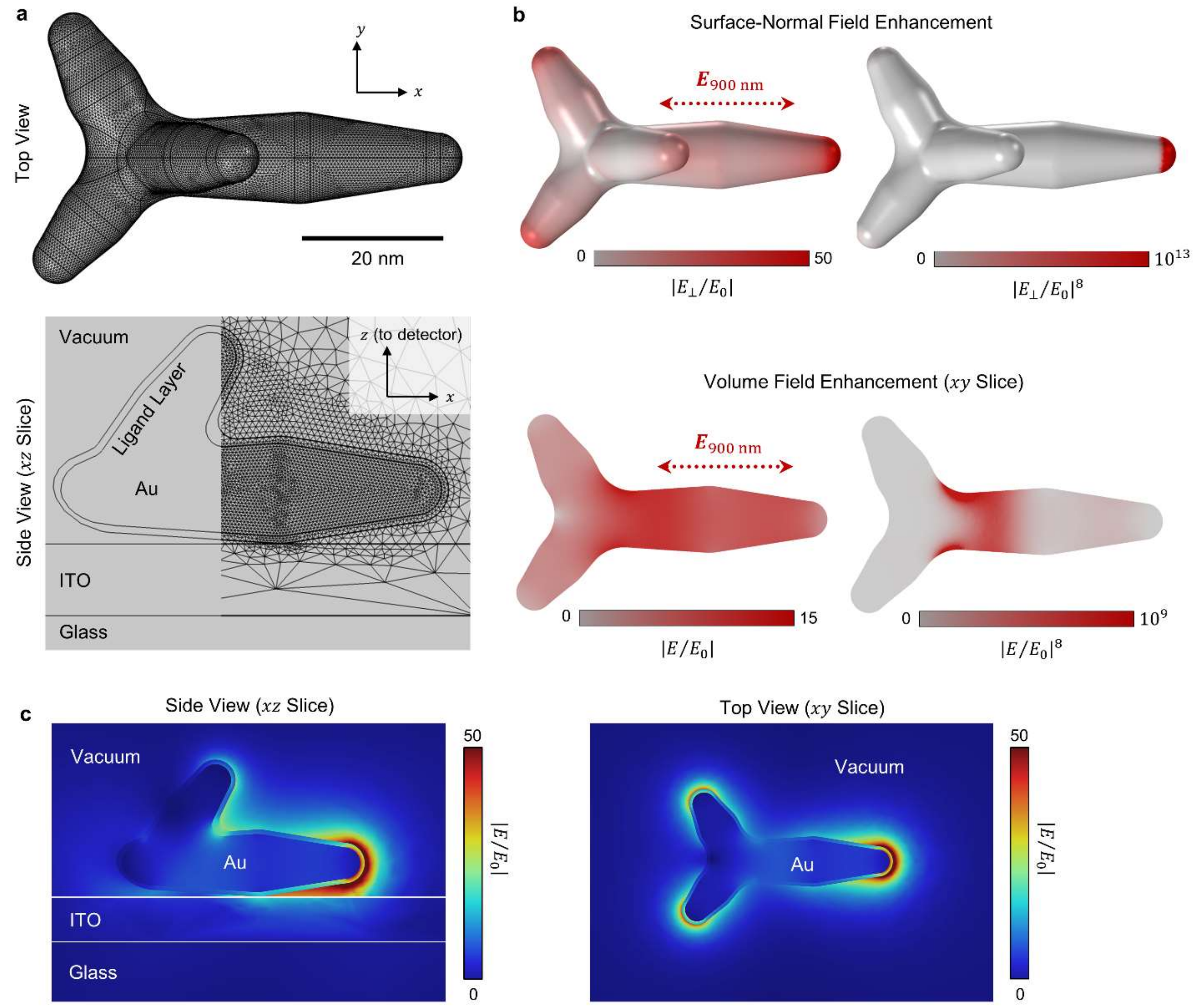

Supplementary Fig. 2 | Finite element simulations. **a** Uniform nanostar surface mesh and cross-section demonstrating the typical domain and meshing. **b** Volume and surface field enhancements calculated for the resonantly-excited nanostar (900 nm, 0°). The 8th-order field enhancement for the 4PPE process leads to a highly-localized surface field enhancement at the tip (leading to tip-aligned directional emission), but a more spread-out volume field enhancement that would produce more isotropic emission if volume-mediated MPPE processes were dominant. **c** Domain slices showing the field enhancement with a different color map for additional clarity.

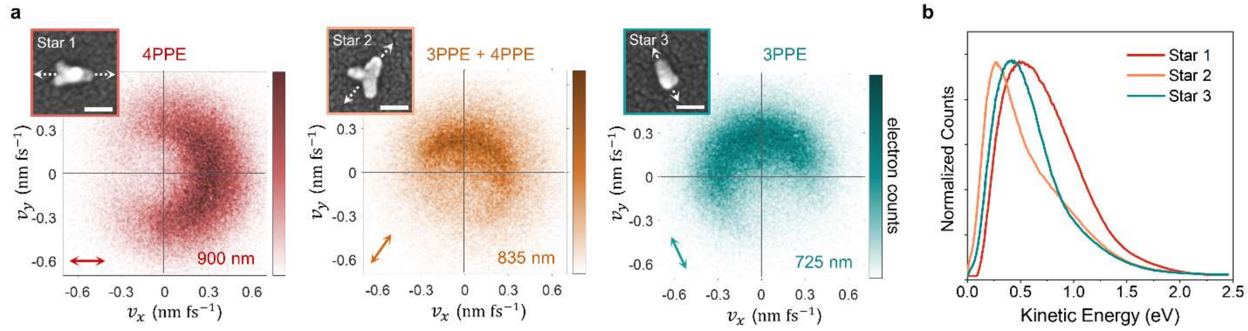

Supplementary Fig. 3 | Single-tip directional velocity distributions. **a** Velocity maps of the three sample stars in the 4PPE regime (Star 1), transition regime (Star 2), and the 3PPE regime (Star 3). The emission is aligned with the resonant tip in each case. All scale bars are 50 nm. **b** Corresponding electron kinetic energy distributions determined from 3D reconstructions, demonstrating a narrowing in the distribution near the onset of the 3PPE regime (e.g. Star 2).

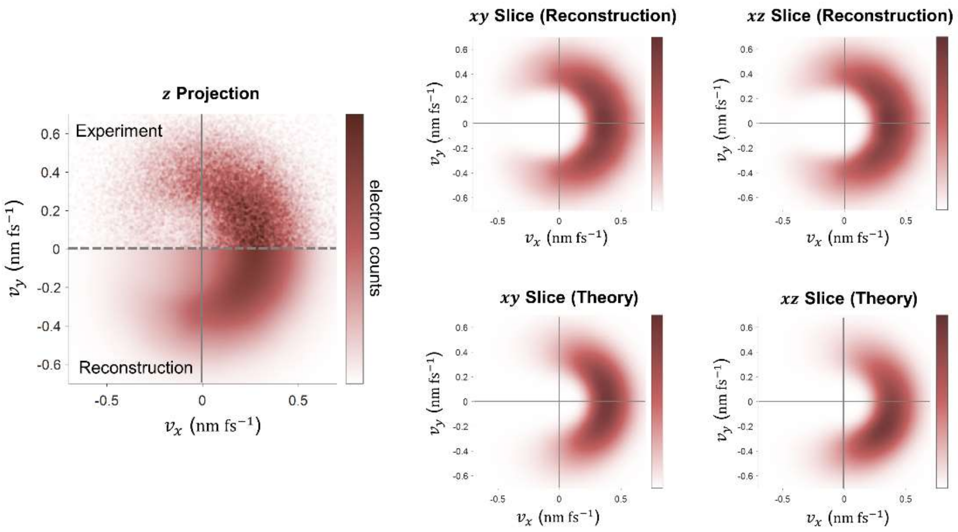

Supplementary Fig. 4 | Experimental and reconstructed velocity distributions. Comparison between experimental and reconstructed photoelectron velocity projections for Star 1, along with slices through the reconstructed and theoretical 3D distributions. The reconstruction is in good agreement with the experimental velocity map image. The theoretical calculations are also in good agreement with the reconstructed/experimental distributions, aside from a slight skew toward the surface ($-z$ direction) in the xz slice due to the induced ITO substrate image charge oscillation.

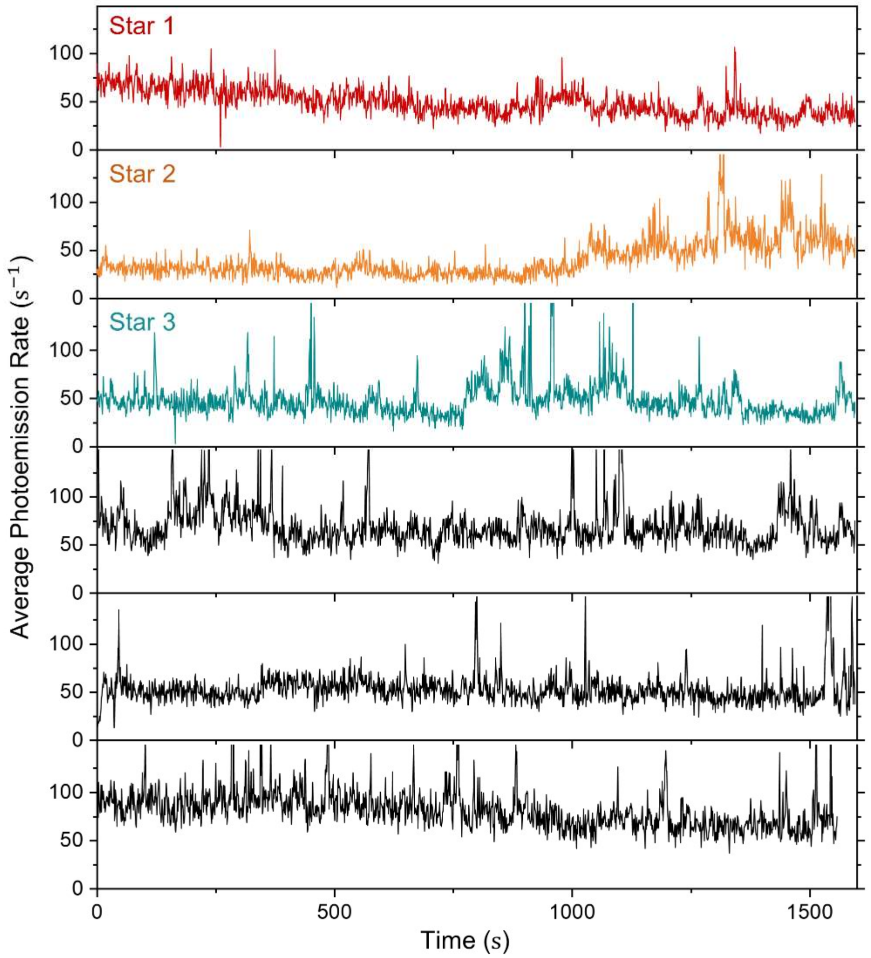

Supplementary Fig. 5 | Photoemission time traces. Average photoemission rate (as opposed to peak rate) as a function of time measured for six nanostar emitters. Each star is a single-tip emitter with well-defined photoemission directionality, as has been demonstrated for representative Stars 1-3. Each time trace therefore represents photoemission from a single nanostar tip. Due to the nonlinear process (MPPE $\propto E^6$ or E^8), small fluctuations in the near-electric-field at the tip ($\pm 10\%$) readily lead to the observed signal fluctuations, although the cause of such near-field fluctuations is not currently known. The average laser power is stable to < 2% during measurements.

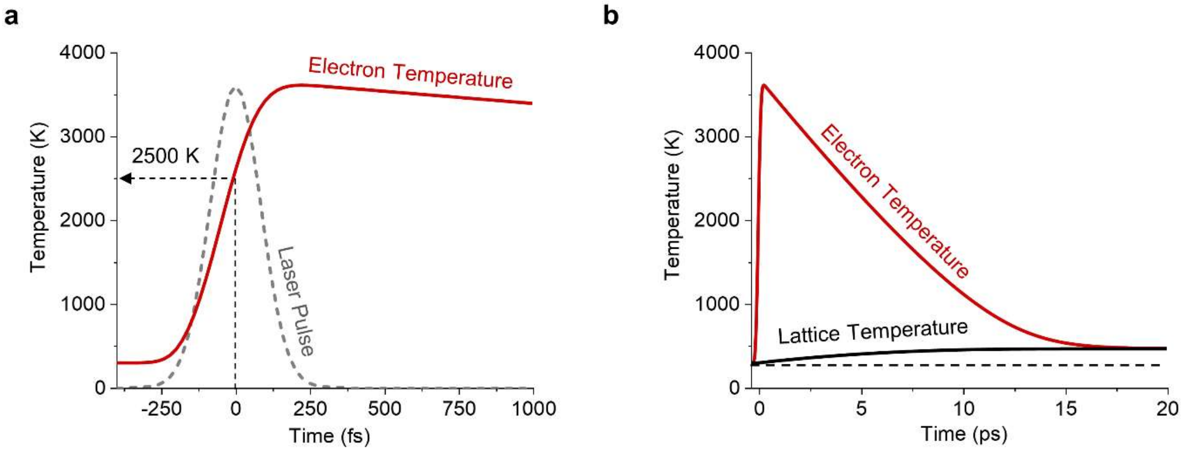

Supplementary Fig. 6 | Electron and lattice heating. **a** Electron temperature calculated via the two-temperature model (Supplementary Note 4) with the parameters utilized for Star 1 measurements and determined via simulations: peak input intensity 2×10^8 W cm^{-2}, 200 fs pulse duration, nanostar volume of 10^4 nm^3, and a linear absorption cross-section of 10^4 nm^2. The effective temperature measured in MPPE experiments can be expected to be approximately the temperature at the pulse peak, i.e. ~2500 K. Due to similar excitation conditions, cross-sections, and volumes for the nanostars studied, this temperature is utilized in all photoemission calculations described in the main text. **b** Evolution of electron and lattice temperatures calculated via the two-temperature model. Peak lattice temperatures less than 500 K are too low to induce nanostar melting.

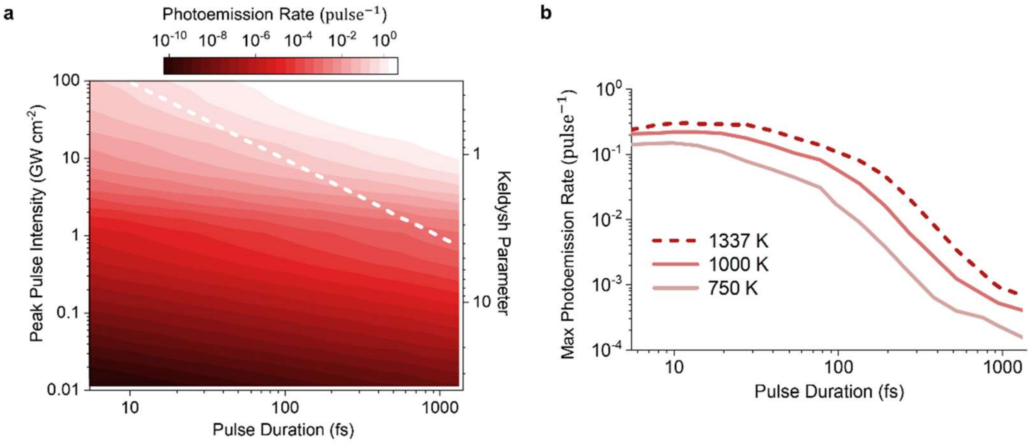

Supplementary Fig. 7 | Nanostar photoemissivity and limitations due to melting. **a** The photocurrent is calculated using the surface photoemission theory as a function of pulse duration and input pulse intensity, assuming 750 nm excitation (3PPE in weak-field regime), 4.5 eV work function, average nanostar volume of 10^4 nm^3, linear absorption cross-section of 10^4 nm^2 and tip field enhancement factor of 50 from simulations. The dashed line represents the upper limit on pulse energy (intensity × duration) at which the onset of particle melting will occur at or around the melting temperature of bulk gold (1337 K). **b** The corresponding maximum photocurrent is plotted as a function of pulse duration for different possible nanostar melting temperatures, as particle melting typically occurs at lower temperatures due to the high fraction of more weakly-bound surface atoms.

Supplementary Note 1

Finite Element Simulations

Simulations are performed in the frequency domain using the RF module in COMSOL Multiphysics 5.4, which is optimized for object sizes comparable to the wavelength of the electromagnetic field. Finite element methods are well-suited to solving for nanoparticle surface field enhancements, as the weak formulation of the problem alleviates one differential order from the equations and thus ameliorates spurious numerical noise at sharp boundaries. This is important for surface-mediated nPPE calculations, in which any spurious noise in the surface field is amplified by the power $2n$. No overall particle symmetry can be assumed for the nanostars, and thus the calculations are conducted in a full 3D domain, which consists of the ITO/glass substrate, vacuum superstrate, gold nanostar with a ligand layer, and a perfectly-matched layer to prevent field reflection at the domain boundaries. A uniform triangular mesh on the nanostar surface (e.g. Supplementary Figure 2) is constructed to be much smaller than any star dimension or electric field variation, and the remainder of the domain is then meshed via combination of tetrahedral elements and rectangular elements. Due diligence is performed on all domain and mesh parameters to ensure convergence in the calculated fields.

Nanostars are modelled in SolidWorks 2017 with a 10 nm diameter core and cylindrically-symmetric arms modelled using dimensions (lengths, widths, and angles) from correlated SEM micrographs, minus the 3 nm Pt coating layer applied prior to imaging. Out-of-plane arms are modelled with shapes/widths determined via TEM statistics (Supplementary Figure 1) and shorter lengths of ~25 nm. The tips are modelled with radii of 3.4 nm based on TEM statistical characterization. A 1 nm HEPES ligand layer surrounds the nanostar, with the refractive index set to 1.5 based on typical values for similar organic molecules in the visible

range. A small extinction coefficient $k_{\text{lig}} = 0.25$ is included for the ligand layer to account for a consistent red-shift in the experimental versus simulated absorption spectra. This is not expected for the HEPES itself, but is instead attributed to amorphous carbon buildup that commonly occurs in nanotip systems in the regions of strong optical field enhancement[1]. Here the carbon buildup may be attributed to photon- or hot electron-mediated cracking and reorganization of the organic ligands. Consistent application of this small extinction coefficient uniformly improves spectral agreement for all calculations, possibly accounting for other damping effects not considered in the modelling. The complex refractive index of the 10 nm ITO film is determined via ellipsometry and the complex refractive index of gold is taken from the literature[2].

Supplementary Note 2

Reconstruction of 3D Photoelectron Velocity Distributions

The v_z projection (forward Abel transform) in velocity map imaging (VMI) experiments is given by

$$\mathrm{VMI}(v_x, v_y) = \int_{\infty}^{\infty} f(v_x, v_y, v_z) dv_z\,, \tag{S1}$$

for initial 3D distribution $f(v_x, v_y, v_z)$. This projection/transform can be reversed for 3D distributions with cylindrical symmetry along an axis parallel to the detector – i.e. $f(v_x, v_y, v_z) = \tilde{f}(v_r, v_y)$ for y-axis cylindrical symmetry – using various inverse Abel transform methods. Here we employ the basis set expansion (BASEX) algorithm of Dribinki *et al.*[3] using the nearly-Gaussian basis set from the paper, with $\sigma = 1$ px and a regularization parameter value of 10. The transform is carried out efficiently via matrix inversion and multiplication in MATLAB, yielding the original 3D photoelectron velocity distribution so long as the cylindrical symmetry assumption is valid, otherwise yielding an approximating to the original distribution.

For the cylindrical symmetry assumption to remain valid for a nanotip supported on a substrate, the surface must either (i) perfectly absorb or (ii) specularly scatter electrons without distorting the velocity distribution – i.e. independent of the incident angle between the electrons and the surface. Whether or not this condition is satisfied depends on the presence of angle-dependent barrier effects, which must be evaluated in a more detailed scattering framework. In addition to these considerations, the otherwise-symmetric field enhancement at the hemispherical tip may become weakly asymmetric due to the ITO image charge formation. This effect is explicitly addressed in the 3D theoretical calculation of Fig. 2f and the xz slice in Supplementary Figure 4. In particular, a slight downward skew toward the substrate is evident in the 3D

distribution, due to the ITO image charge formation. However, the otherwise excellent agreement between reconstruction and theory, particularly in angular/energy distributions in Fig. 2 of the main text, indicates that this effect is relatively small.

Supplementary Note 3

Nanostar Spatiotemporal Coherence

The temporal coherence is primarily limited by the photoelectron kinetic energy spread, leading to longitudinal dispersion that is most prominent immediately following emission (prior to acceleration in the electrostatic lens), when the average electron kinetic energy is comparable to the spread in the energy distribution. A full width at half maximum kinetic energy spread around 0.8 eV is observed for the characteristic nanostar tip MPPE distribution measured in Fig. 2 of the main text. This is a substantial improvement over typical kinetic energy spreads >10 eV in the optical field emission regime[4,5] and can be improved further by carefully matching the n-photon energy to the work function[6]. The transverse spatial coherence is often characterized via the normalized root-mean-square beam emittance, a conserved phase space quantity in aberration-free systems defined by

$$\varepsilon_{\text{rms}} = \beta \gamma_L \sigma_r \sigma_\theta, \tag{S2}$$

at the beam waist[7], in which $\beta = v/c$ for average photoelectron velocity v, γ_L is the Lorentz factor, σ_r is the transverse spatial uncertainty, and σ_θ is the angular uncertainty. Since a beam waist is located at the source, the emittance is approximated via the tip size ($\sigma_r = 3.4$ nm, Supplementary Figure 1), angular emission uncertainty ($\sigma_\theta = 70° = 1220$ mrad, e.g. Fig. 2g), and average velocity ($v/c = 0.0014$), as 5.9 nm mrad. This serves as an upper limit, as the effective source size is determined by tracing electron trajectories back to the center of the hemispherical tip[8]. Even as an upper limit, this nanostar tip MPPE beam emittance is approaching values achieved on pulsed nanotip sources (around 1.7 nm mrad) in the linear photoemission regime[9], which, in turn, are only an order of magnitude higher than the Heisenberg uncertainty limit of $\varepsilon_{\text{rms}} = \hbar/(2m_e c) = 0.19$ nm mrad.

Nanostar Brightness Limits

Limits on the achievable photoemission currents from gold nanostars and other plasmonic nanoparticles are imposed either by space-charge effects or by particle melting at high excitation pulse energies. It is shown in Supplementary Figure 7 that nanostar melting is expected to occur at photocurrents less than 1 electron/pulse, which precludes any space-charge effects. We do not approach these melting limits in the present studies and are therefore far from the regime in which space-charge effects become important. Photocurrents exceeding 100 electrons emitted in a femtosecond pulse have, however, been demonstrated for patterned gold nanorods[10], in which case space-charge limitations do become important. The peak photocurrents reported in Supplementary Figure 7 are calculated using the surface MPPE theory presented in the main text. The peak nanostar lattice temperatures are calculated simply from the bulk heat capacity of gold, a typical nanostar volume of 10^4 nm^3, and simulated linear absorption cross-section of 10^4 nm^2 (Supplementary Note 3). The bulk gold melting temperature (1337 K) serves as an upper limit on the nanostar melting temperature, although nanostar melting is likely to occur at lower temperatures due to the high nanoparticle surface-to-volume ratio and more weakly-bound surface atoms. It is evident that higher intensities can be utilized with shorter pulse durations due to the trade-off between linear absorption and third-order photoemission cross-sections, but the effective photoemission order and the maximum achievable photocurrent begin to level off at shorter pulse durations as the Keldysh parameter drops below unity and into the optical field emission regime.

Supplementary Note 4

Two-Temperature Heating Model

Electron and phonon (lattice) temperatures are calculated for gold nanostars following pulsed excitation using the two-temperature model[11]. Excited plasmons decay into electron-hole pairs, leading to heating of the electron gas (Supplementary Figure 6a), which thermalizes via electron-electron scattering on ~100 fs timescales before any substantial energy transfer to nanoparticle lattice occurs. Electron-lattice thermalization occurs on few-picosecond timescales via electron-phonon scattering (Supplementary Figure 6b). Approximating the pulse energy to be directly transferred into the thermalized electron distribution, the coupled two-temperature equations are

$$C_e(T_e)\frac{dT_e}{dt} = -g(T_e - T_\mathrm{l}) + I(t)\sigma_\mathrm{abs}, \quad \text{(S3a)}$$

$$C_\mathrm{l}\frac{dT_\mathrm{l}}{dt} = g(T_e - T_\mathrm{l}), \quad \text{(S3b)}$$

with electron-phonon coupling constant[11,12] $g = 2\times10^{-7}$ W K^{-1} , Gaussian pulse intensity $I(t)$, nanostar absorption cross-section $\sigma_\mathrm{abs} \approx 10^4$ nm^2, electron heat capacity C_e, and lattice heat capacity C_l. The temperature-dependent free-electron Sommerfeld heat capacity[13] is $C_e(T_e) = \pi^2 k_\mathrm{B}^2 T_e n_e/(2E_\mathrm{F}) = 1.5\times10^{-19}$ J K^{-1}, with free electron density $n_e = 5.9\times10^{28}$ m^{-3} and Fermi energy $E_\mathrm{F} = 5.53$ eV for gold. The lattice heat capacity, $C_\mathrm{l} = c_\mathrm{l}\rho V = 2.5\times10^{-17}$ J K^{-1}, is calculated using the specific heat of bulk gold, $c_\mathrm{l} = 129$ J $\mathrm{K}^{-1}\mathrm{kg}^{-1}$, density of gold $\rho = 19.32$ kg m^{-3}, and nanoparticle volume $V = 10^4$ nm^3. The lattice heat capacity is over two orders of magnitude larger than the electron heat capacity, which explains the much lower peak lattice temperature (~500 K) compared with the peak electron temperature (~2500 K).

Supplementary References

1 Szczerbinski, J., Gyr, L., Kaeslin, J. & Zenobi, R. Plasmon-driven photocatalysis leads to products known from e-beam and x-ray-induced surface chemistry. *Nano Lett.* **18**, 6740-6749 (2018).

2 Johnson, P. B. & Christy, R. W. Optical constants of noble metals. *Phys. Rev. B* **6**, 4370-4379 (1972).

3 Dribinski, V., Ossadtchi, A., Mandelshtam, V. A. & Reisler, H. Reconstruction of abel-transformable images: The gaussian basis-set expansion abel transform method. *Rev. Sci. Instrum.* **73**, 2634-2642 (2002).

4 Dombi, P. *et al.* Ultrafast strong-field photoemission from plasmonic nanoparticles. *Nano Lett.* **13**, 674-678 (2013).

5 Kruger, M., Schenk, M. & Hommelhoff, P. Attosecond control of electrons emitted from a nanoscale metal tip. *Nature* **475**, 78-81 (2011).

6 Aidelsburger, M., Kirchner, F. O., Krausz, F. & Baum, P. Single-electron pulses for ultrafast diffraction. *Proc. Natl. Acad. Sci. USA* **107**, 19714-19719 (2010).

7 Reiser, M. *Theory and design of charged particle beams*. (WILEY-VCH Verlag GmbH & Co. KGaA, 2008).

8 Ehberger, D. *et al.* Highly coherent electron beam from a laser-triggered tungsten needle tip. *Phys. Rev. Lett.* **114**, 227601 (2015).

9 Feist, A. *et al.* Ultrafast transmission electron microscopy using a laser-driven field emitter: Femtosecond resolution with a high coherence electron beam. *Ultramicroscopy* **176**, 63-73 (2017).

10 Hobbs, R. G. *et al.* High-yield, ultrafast, surface plasmon-enhanced, Au nanorod optical field electron emitter arrays. *ACS Nano* **8**, 11474-11482 (2014).

11 Hartland, G. V. Optical studies of dynamics in noble metal nanostructures. *Chem. Rev.* **111**, 3858-3887 (2011).

12 Fann, W. S., Storz, R., Tom, H. W. K. & Bokor, J. Electron thermalization in gold. *Phys. Rev. B* **46**, 13592-13595 (1992).

13 Ashcroft, N. W. & Mermin, N. D. *Solid state physics*. (Saunders College, 1976).
